# VOLUNTEER ACTIVITY CHANGES AFTER MIDDLE YEARS OLD AND THE INFLUENCES OF VOLUNTEERING ON WELL-BEING

**DOI:** 10.1093/geroni/igz038.634

**Published:** 2019-11-08

**Authors:** Hyunkee Lee

**Affiliations:** Korean Gerontological Society, Suwon, South Korea, Korea, Republic of

## Abstract

The objectives of this paper are to examine volunteer activity changes over the life courses and the long-term influences of volunteer activity on the well-being. The paper analyzes the KLoSA data from wave 1 to wave 5, and selects 10,254 persons over 45 years old as study samples in the base year. For the statistical analysis, the two-way connected line plot and transition probability analysis techniques are employed and the GEE method is used for the multivariate regression estimation of the coefficients. The results show that the volunteer activities increase highest in one’s late 40s and decrease abruptly from the age, and slow down 50s to one’s late 70s, showing a repeating rise and fall pattern. Entering one’s 80s, the volunteer activities come to be stable relatively than the previous years. And, the probability of continuing the volunteer status after 2 years is about 31.2 percent and the probability of stopping volunteer activity comes to be around 68.9 percent. As a last, the regression analysis results show that there is a positive relationship between volunteerism and well-being variables. Expecially the interaction term is statistically significant, showing a negative sign of the coefficient. This implies that the volunteerism contributes to increasing the well-being but decreasing by engaging excessively. The results support the previous study outcomes that excessive volunteer activities are not good for the health.

